# Impact of a Social Robot on Hospitalized Children, Caregivers, and Health Care Staff: Exploratory Observational Study

**DOI:** 10.2196/93897

**Published:** 2026-07-10

**Authors:** Atsushi Iwai, Naohiro Matsumaru, Minami Ohshima, Ibuki Yasui, Akane Yoshida, Ayumi Ohno, Hiroyuki Kuroda, Kuniaki Tanaka, Kenichiro Kobayashi, Takako Tachibana, Ikuya Usami, Yusuke Ito, Toshiro Maihara

**Affiliations:** 1 Department of Pediatric Hematology and Oncology, Hyogo Prefectural Amagasaki General Medical Center, 2-17-77 Higashinaniwa-cho, Amagasaki, Hyogo, 660-8550, Japan, 81 06-6480-7000, 81 06-6480-7001; 2 Department of Pediatrics, Hyogo Prefectural Amagasaki General Medical Center, Amagasaki, Hyogo, Japan; 3 Department of Nursing, Hyogo Prefectural Amagasaki General Medical Center, Amagasaki, Hyogo, Japan; 4 Department of Pediatric Emergency and Critical Care, Hyogo Prefectural Amagasaki General Medical Center, Amagasaki, Hyogo, Japan

**Keywords:** social robot, companion robot, pediatric hospitalization, psychosocial intervention, LOVOT, child health, caregivers, health care staff

## Abstract

**Background:**

Hospitalization poses significant psychosocial challenges for children, including anxiety, emotional distress, and disruption of daily routines. While animal-assisted therapy has shown benefits, practical barriers such as infection control concerns and inconsistent availability limit its implementation in pediatric wards. Social robots have emerged as a promising alternative, but evidence on their psychosocial impact in pediatric settings remains limited. LOVOT is a companion robot designed for emotional engagement through warmth, softness, and nonverbal interaction; however, no studies have examined its effects in pediatric health care.

**Objective:**

This exploratory study aimed to assess the perceived impact of LOVOT on hospitalized children, their caregivers, and health care staff in a pediatric ward.

**Methods:**

This prospective observational study was conducted in a pediatric ward at Hyogo Prefectural Amagasaki General Medical Center, Japan, from May 2024 to March 2025. Two LOVOT units were permanently installed in the playroom for free interaction. Caregivers (n=110) and health care staff (n=32) completed postintroduction questionnaires with retrospective ratings of perceived change using 5-point Likert scales (1=“much worse” to 5=“much better,” with 3=“no change” as the baseline). Free-text responses (n=91) were analyzed using automated emotion classification. One-sample 2-tailed *t* tests or Wilcoxon signed-rank tests compared ratings against the baseline, with false discovery rate (FDR) correction for multiple comparisons.

**Results:**

All 10 caregiver-rated and 11 of 12 staff-rated outcomes showed significant perceived improvement (all caregiver items FDR *P*<.001; staff items FDR *P*≤.02; the workload item was not significant, *P*=.37). Mean ratings ranged from 3.28 to 4.62 on the 5-point scale (baseline: 3=“no change”). The largest perceived improvements were reported for children’s enjoyment of hospital life (mean 4.47, SD 0.62), adaptation to hospital life (mean 4.10, SD 0.70 to mean 4.28, SD 0.63), and stress and anxiety reduction (mean 4.17, SD 0.76 to mean 4.19, SD 0.64). One-sample effect sizes ranged from medium to very large (Cohen *d*=0.53-2.67); however, because these were calculated against a fixed neutral baseline rather than a control group, they should be interpreted cautiously. Ratings from caregivers and staff showed strong agreement on 4 of 5 matched items. Notably, no detectable increase in staff workload was observed (mean 3.09, SD 0.53; *P*=.37), supporting implementation feasibility. Qualitative analysis revealed predominantly positive responses, with joy as the dominant emotion (mean probability 88.7%, 95% CI 86.8%-90.6%).

**Conclusions:**

This study provides preliminary evidence that a companion robot (LOVOT) may positively impact hospitalized children, caregivers, and health care staff across multiple psychosocial outcomes without a detectable increase in staff workload. The low response rate and reliance on retrospective self-report limit generalizability and causal inference; however, convergent findings across stakeholder perspectives justify further investigation through controlled trials. Companion robots represent a feasible, low-burden adjunct to psychosocial care in pediatric settings.

## Introduction

Hospitalization represents a significant source of stress for children and their families. Children may experience anxiety, fear, and emotional distress due to unfamiliar environments, separation from home, painful procedures, and disruption of daily routines [1,2]. These negative experiences can impair recovery, increase behavioral problems, and produce lasting psychological effects [3]. Caregivers also experience substantial stress when their children are hospitalized, which can affect their own well-being and their ability to support their children [2,4].

Various interventions have been developed to address the psychosocial needs of hospitalized children. Play therapy and child life programs are well-established approaches that use play to reduce anxiety and facilitate coping [5-7]. Animal-assisted therapy has also shown promise in reducing stress and improving mood in pediatric settings [8,9]. However, animal-assisted therapy faces practical limitations including infection control concerns, animal welfare considerations, allergies, and inconsistent availability [10,11].

Social robots have emerged as a potential alternative or complement to existing interventions. These robots are designed to interact with humans in socially meaningful ways and have been studied in various health care contexts [12,13]. In pediatric settings, social robots such as Paro (a therapeutic seal robot) and humanoid robots have demonstrated positive effects on children’s emotional states and engagement [14-17]. Social robots offer practical advantages over live animals, including consistent availability, no infection risk, and standardized interaction quality.

LOVOT is a companion robot developed in Japan that differs from most social robots studied in health care. Rather than performing tasks or engaging in verbal communication, LOVOT is designed exclusively for emotional engagement through nonverbal interaction, featuring warmth, softness, and an endearing appearance that encourages physical contact such as holding and stroking [18]. To date, no studies have examined LOVOT’s effects in pediatric health care settings.

The aim of this study was to assess the perceived impact of LOVOT on hospitalized children, their caregivers, and health care staff in a pediatric ward. We examined perceived changes in psychosocial outcomes following LOVOT introduction using retrospective questionnaires administered to caregivers and staff complemented with qualitative analysis of free-text responses.

## Methods

### Study Design and Setting

This prospective observational study was conducted at the Department of Pediatrics, Hyogo Prefectural Amagasaki General Medical Center, Japan, between May 2024 and March 2025.

### Participants

Caregivers of hospitalized children who used the pediatric playroom during the study period were eligible to participate. Questionnaires were available in the playroom for voluntary completion. Caregivers who answered “No” to the question asking whether they had any contact with LOVOT were excluded from analysis. Children’s demographic and clinical characteristics were reported by their caregivers. During the study period, approximately 2500 children were admitted to the pediatric ward, of whom an estimated 25% used the playroom (approximately 625 children); however, the exact number of unique playroom users was not systematically recorded because no check-in system was in place. The estimated response rate was 18.4% (115/625), although both the numerator (respondents) and denominator (playroom users) involve uncertainty, and the true response rate may differ. All health care professionals working in the pediatric ward were invited to participate.

### LOVOT Intervention

**Figure 1.**
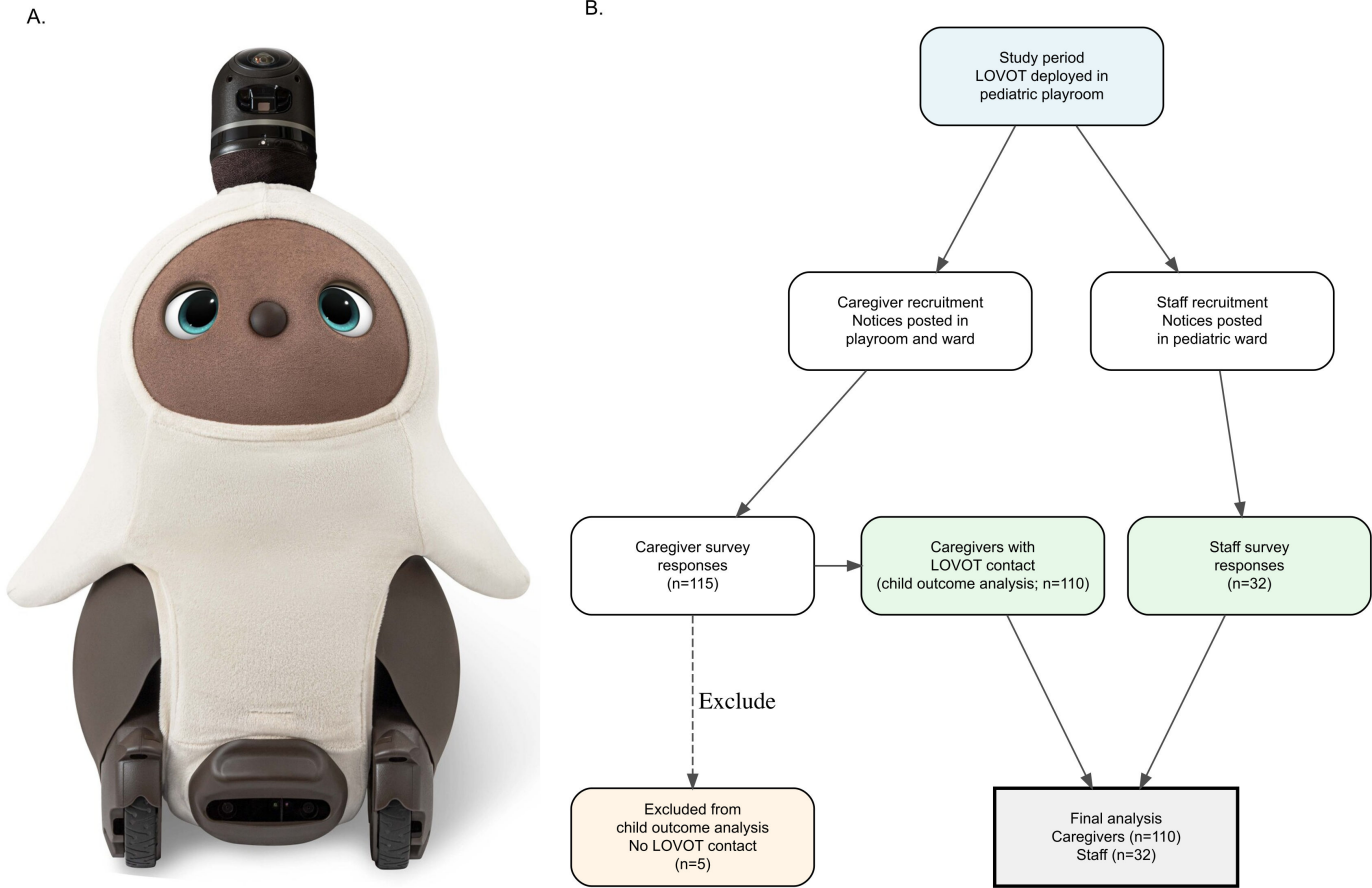
Study overview: (A) LOVOT companion robot used in the study and (B) study flow diagram showing participant recruitment and exclusion criteria.

Two LOVOT 2.0 units (Groove X, Inc) were permanently installed in the pediatric playroom, enabling free interaction among hospitalized children, families, and health care staff throughout the study period (Figure 1A). LOVOT is designed for nonverbal communication through physical contact, featuring a warm body temperature (approximately 37 °C), a soft tactile exterior, and an endearing appearance that encourages holding and stroking (detailed technical specifications are provided in Multimedia Appendix 1).

Health care staff received a brief orientation on LOVOT handling and basic maintenance (charging and cleaning); however, no structured intervention program or protocol was implemented. This approach was chosen to maintain naturalistic observation conditions and assess LOVOT’s effects in real-world clinical practice. For infection control, LOVOT’s removable clothing was changed and laundered every 2 to 3 days, and the surface was cleaned daily with alcohol-containing disinfectant wipes. LOVOT required approximately 15 minutes of charging per hour; during charging periods, children could still interact with LOVOT and often enjoyed observing it in a “sleeping” state.

### Data Collection

Caregivers completed a self-administered postintroduction questionnaire with retrospective ratings of perceived change using 5-point Likert scales (1=“much worse,” 2=“somewhat worse,” 3=“no change,” 4=“somewhat better,” and 5=“much better”). No baseline assessment was conducted prior to LOVOT introduction. A study-specific questionnaire was developed because no validated instrument existed for assessing the perceived impact of companion robots in pediatric settings. Existing measures of child distress or anxiety (eg, the Child Health Questionnaire) assess current states rather than perceived change attributable to a specific intervention, and general technology acceptance scales do not capture the psychosocial domains relevant to this study. Questionnaire items were drafted based on clinically relevant domains identified from the social robotics literature [12,13,19] and reviewed for content and face validity by the multidisciplinary research team, including pediatricians, nurses, and childcare workers. Internal consistency was assessed post hoc using the Cronbach α; however, formal psychometric validation, including test-retest reliability and construct validity, was not conducted. The questionnaire included 10 items assessing child outcomes (7 items) and caregiver outcomes (3 items), along with demographic information and free-text responses (Table S1 in Multimedia Appendix 2).

Staff completed a questionnaire using the same 5-point Likert scale, including 12 items assessing impact on children and caregivers (7 items) and impact on staff and ward environment (5 items), along with demographic information (Table S2 in Multimedia Appendix 2).

### Statistical Analysis

All statistical analyses were conducted using R (version 4.5.1; R Foundation for Statistical Computing). Python (version 3.13.7; Python Software Foundation) was used for qualitative text analysis. All analysis code is publicly available [20] under the MIT License.

No primary outcome was prespecified; all analyses were exploratory. For quantitative data, 1-sample tests compared ratings against the baseline value (3=“no change”), with normality assessed using the Shapiro-Wilk test. 2-tailed *t* tests were used for normally distributed data; Wilcoxon signed-rank tests were used for nonnormally distributed data. The Benjamini-Hochberg procedure controlled the false discovery rate (FDR; value of <.05). Effect sizes were calculated using the Cohen *d* from 1-sample tests (mean difference from baseline divided by sample SD), interpreted as small (0.2), medium (0.5), large (0.8), very large (1.2), and huge (≥2.0; [21]). Because these 1-sample effect sizes compared ratings against a fixed neutral baseline rather than a control group mean, they were expected to be larger than between-group effect sizes from controlled designs and should not be directly compared to effect sizes from randomized controlled trials. Likert ratings were treated as interval data for this purpose; the resulting Cohen *d* values should be read as indexes of the magnitude of departure from the neutral midpoint, not as standardized between-group differences.

The internal consistency of the questionnaire subscales was assessed using the Cronbach α, calculated separately for child outcomes (7 items) and caregiver outcomes (3 items) on the caregiver questionnaire and impact on children and caregivers (7 items) and impact on staff and ward environment (4 items) on the staff questionnaire (workload was excluded from the latter subscale because it measures a conceptually distinct construct with a different scale direction).

Subgroup analyses stratified children’s outcomes by age (≤5 vs ≥6 years), hospital stay duration (≤1 week vs >1 week), sex, and playroom use frequency. Caregiver and staff ratings were compared using Mann-Whitney *U* tests for 5 matched items. Missing data were handled on an item-by-item basis, with available responses analyzed for each variable.

For qualitative data, Japanese free-text responses were tokenized using the MeCab morphological analyzer with the fugashi library. A domain-specific stop word list (96 terms; Table S1 in Multimedia Appendix 3) was applied, and the top 20 high-frequency terms were visualized using horizontal bar charts, with Japanese-English translations provided in Table S2 in Multimedia Appendix 3. Emotion classification was performed using the LUKE-Japanese-large model fine-tuned on the WRIME dataset (a Japanese emotion corpus with multi-rater annotations), classifying responses into 8 emotions (joy, sadness, anticipation, surprise, anger, fear, disgust, and trust). Bootstrap 95% CIs were calculated with 5000 iterations.

Detailed statistical methods are provided in Multimedia Appendix 1.

### Ethical Considerations

This study was conducted in accordance with the Declaration of Helsinki and approved by the Institutional Review Board of Hyogo Prefectural Amagasaki General Medical Center (approval number: 6-58). Informed consent was obtained from all participants through completion of a consent checkbox on the questionnaire, a method approved by the institutional review board given the anonymous, noninvasive nature of the survey. All data were collected anonymously; no personally identifiable information was recorded. Children’s demographic and clinical characteristics were reported by their caregivers in aggregate form. Free-text responses were analyzed without any identifying information.

## Results

### Participant Characteristics

Of 115 caregiver respondents, 5 (4.3%) were excluded based on self-reported absence of LOVOT contact, resulting in a final analytical sample of 110 (95.7%) caregivers (Figure 1B). A total of 32 health care staff members completed the survey. Caregiver respondents were predominantly female (82/93, 88.2%), and the median child age was 5 (IQR 3-8) years. Staff comprised 45.2% (14/31) physicians, 48.4% (15/31) nurses, 6.5% (2/31) childcare workers; occupation was not reported by 1 staff member (Table 1). The children had hospital stay durations of 1 week or less (73/106, 68.9%), 1 week to 1 month (21/106, 19.8%), or 1 month or more (12/106, 11.3%). Most children used the playroom frequently (at least once per day: 66/105, 62.9%). Sex and age data were complete for all children (n=110); 48.2% (53/110) were male and 51.8% (57/110) were female. No adverse events related to LOVOT interaction, including injuries, falls, or choking hazards, were reported by caregivers or staff through spontaneous reporting during the study period, and no systematic adverse event monitoring protocol was implemented.

**Table 1.** Participant (caregiver and child) and health care staff characteristics.

Characteristic	Values
Caregivers and children (n=110)	
Child age (years), median (IQR)	5.0 (3.0-8.0)
Child sex, n/N (%)	
Male	53/110 (48.2)
Female	57/110 (51.8)
Hospital stay duration, n/N (%)	
≤1 week	73/106 (68.9)
1 week to 1 month	21/106 (19.8)
≥1 month	12/106 (11.3)
Playroom use frequency, n/N (%)	
At least once per day	66/105 (62.9)
Approximately once every 2 days	29/105 (27.6)
Less than once every 3 days	10/105 (9.5)
Caregiver age (years), n/N (%)	
≤29	6/107 (5.6)
30-39	63/107 (58.9)
40-49	37/107 (34.6)
50-59	1/107 (0.9)
Caregiver sex, n/N (%)	
Male	11/93 (11.8)
Female	82/93 (88.2)
Health care staff (n=32), n/N (%)	
Occupation	
Physician	14/31 (45.2)
Nurse	15/31 (48.4)
Childcare worker	2/31 (6.5)
Age (years)	
20-29	15/31 (48.4)
30-39	7/31 (22.6)
40-49	4/31 (12.9)
50-59	2/31 (6.5)
60-69	3/31 (9.7)
Sex	
Male	11/31 (35.5)
Female	20/31 (64.5)
Frequency of LOVOT contact	
At least once per day	4/32 (12.5)
Approximately once every 2–3 days	5/32 (15.6)
Approximately once per week	9/32 (28.1)
Approximately once per month	7/32 (21.9)
Rarely or never	7/32 (21.9)

### Questionnaire Internal Consistency

The Cronbach α values indicated acceptable to good internal consistency for the questionnaire subscales (child outcomes on the caregiver questionnaire [7 items]: Cronbach α=0.77; caregiver outcomes on the caregiver questionnaire [3 items]: Cronbach α=0.67; impact on children and caregivers on the staff questionnaire [7 items]: Cronbach α=0.84; impact on staff and ward environment on the staff questionnaire [4 items]: Cronbach α=0.77). The lower Cronbach α value for caregiver outcomes on the caregiver questionnaire reflects the small number of items (3 items); the mean interitem correlation for this subscale was 0.40 (SD 0.07), within the recommended range of 0.20 to 0.50.

### Impact on Children and Caregivers

**Figure 2.**
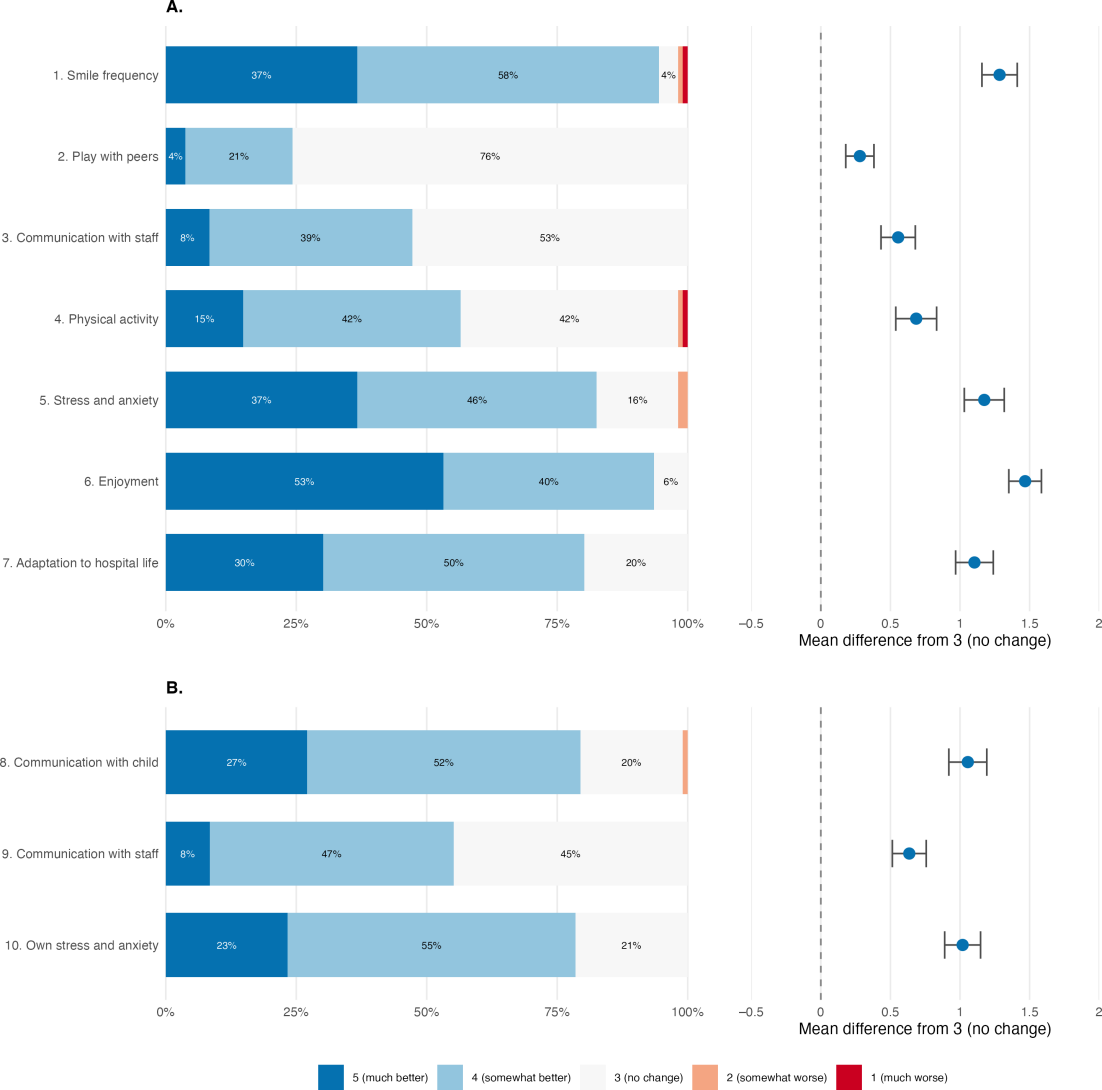
Caregiver-reported outcomes following LOVOT introduction (n=110): (A) children outcomes (items 1-7) and (B) caregiver outcomes (items 8-10). Left panels show the distribution of Likert-scale responses (1-5). Right panels show mean differences from baseline (3=“no change”) with 95% CIs. All items showed statistically significant perceived improvement (false discovery rate *P*<.001). Statistical test results are shown in Table S1 in Multimedia Appendix 4.

All 10 caregiver-rated items showed statistically significant perceived improvement after LOVOT introduction (*P*<.001 in all cases, FDR corrected; Figure 2; Table S1 in Multimedia Appendix 4). These ratings reflect caregivers' perceived change relative to the neutral midpoint (3=“no change”) and should be interpreted as perceived improvements rather than objectively measured effects. One-sample effect sizes (Cohen *d*) ranged from 0.53 to 2.38, indicating medium to very large effects by conventional benchmarks, although these 1-sample estimates should be interpreted cautiously as they are not directly comparable to between-group effect sizes from controlled trials. The largest perceived improvements were reported for children’s enjoyment of hospital life (*d*=2.38; mean 4.47, SD 0.62), stress and anxiety reduction (*d*=1.55; mean 4.17, SD 0.76), and adaptation to hospital life (*d*=1.57; mean 4.10, SD 0.70). Caregivers also reported reduced personal stress and anxiety (*d*=1.51; mean 4.02, SD 0.67). Detailed results for all items are provided in Table S1 in Multimedia Appendix 4.

### Consistency Across Patient Characteristics

**Figure 3.**
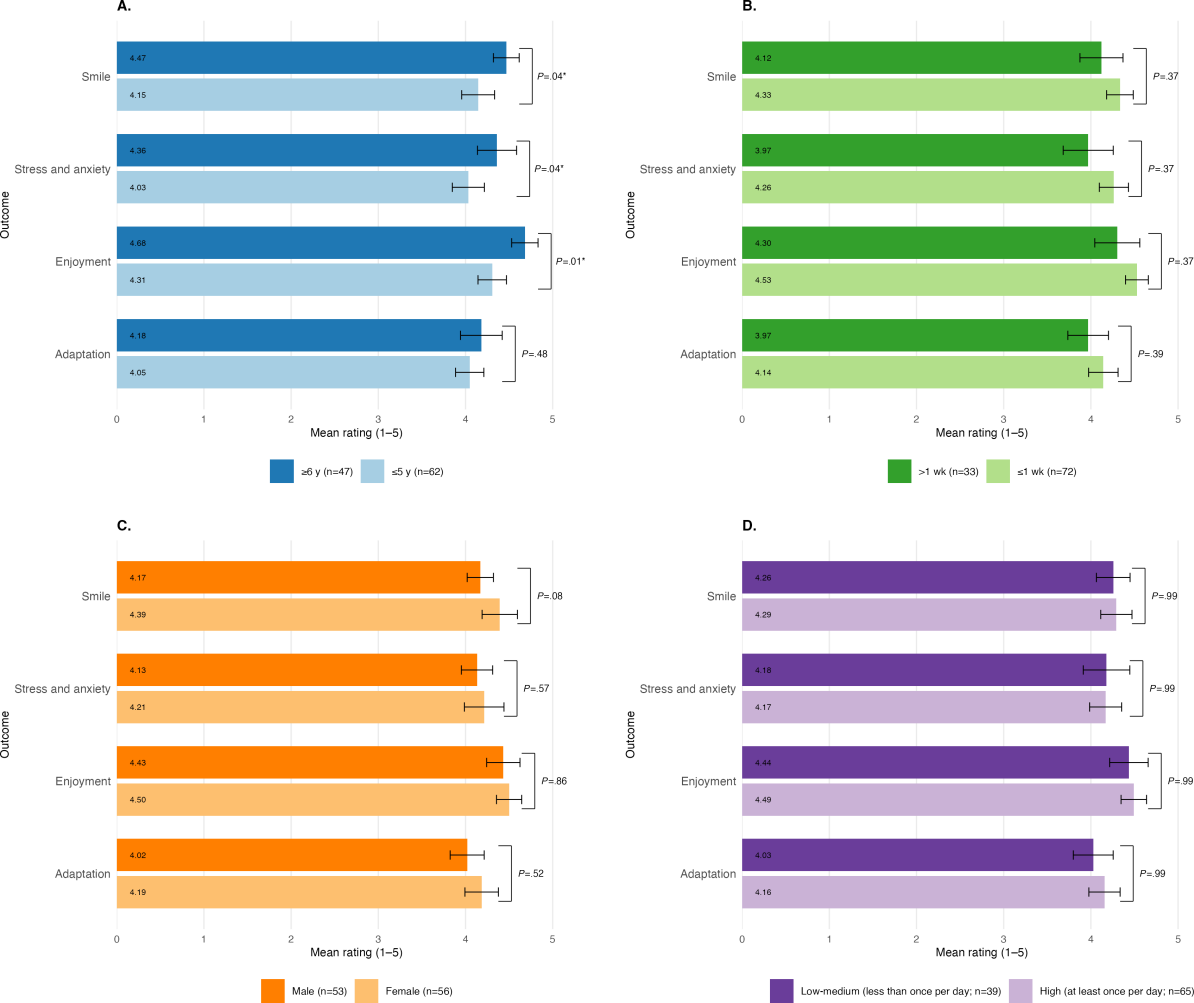
Subgroup analysis of children’s outcomes. Comparison of effects across (A) age groups (≤5 vs ≥6 years), (B) hospital stay duration (≤1 week vs >1 week), (C) sex, and (D) playroom use frequency (at least once per day vs less than once per day). The bars represent mean ratings (scale from 1-5) with 95% CIs. Between-group comparisons were conducted using Mann-Whitney *U* tests with false discovery rate correction (see Table S2 in Multimedia Appendix 4 for complete results). *P<.05.

Subgroup analyses revealed consistent perceived improvements across all examined patient characteristics (Figure 3; Table S2 in Multimedia Appendix 4). All subgroups showed mean ratings of more than 3 (no change) with *P*<.001 for within-group comparisons (FDR corrected). Between-group comparisons showed no significant differences for most items across hospital stay duration (≤1 week vs >1 week), sex, and playroom use frequency (at least once per day vs less than once per day), indicating broad applicability. For age groups, older children (≥6 years) showed significantly higher ratings than younger children (≤5 years) for smile frequency (*P*=.04), stress and anxiety reduction (*P*=.04), and enjoyment (*P*=.01), suggesting that school-aged children may be particularly responsive to LOVOT interaction; however, both age groups showed significant improvements overall.

### Health Care Staff Perspectives

**Figure 4.**
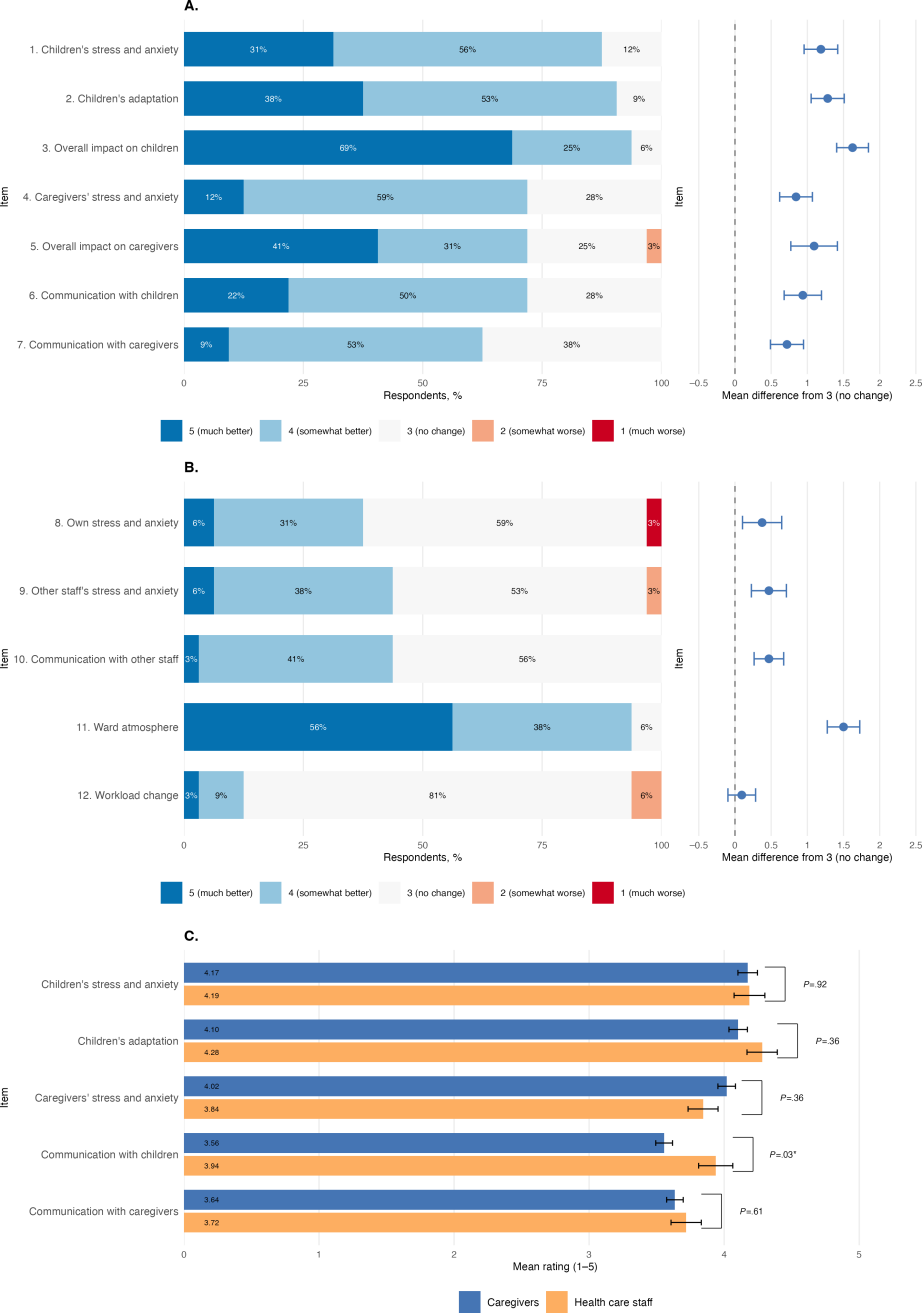
Health care staff perspectives on LOVOT impact (n=32): (A) impact on children and caregivers (items 1-7), (B) impact on staff and ward environment (items 8-12), and (C) comparison between caregiver (n=110) and staff (n=32) ratings for 5 matched items. Left panels show the distribution of Likert-scale responses (1-5). Right panels show mean differences from baseline (3=“no change”) with 95% CIs. The bars represent means with SEs. Between-group comparisons were conducted using Mann-Whitney *U* tests with false discovery rate correction. Statistical test results are shown in Table S3 in Multimedia Appendix 4. *P<.05.

Staff ratings demonstrated significant perceived improvements for 11 of 12 items (Figure 4; Table S3 in Multimedia Appendix 4). One-sample effect sizes ranged from 0.18 to 2.67. The largest perceived improvements were observed for overall impact on children (*d*=2.67; mean 4.62, SD 0.61), ward atmosphere (*d*=2.41; mean 4.50, SD 0.62), and children’s adaptation (*d*=2.02; mean 4.28, SD 0.63). Staff also reported perceived improvements in children’s stress and anxiety (*d*=1.84; mean 4.19, SD 0.64) and caregivers’ stress and anxiety (*d*=1.34; mean 3.84, SD 0.63). Notably, no detectable increase in workload was observed (mean 3.09, SD 0.53; *d*=0.18; *P*=.37, FDR corrected), although the small sample size (n=32) limits statistical power to detect small to medium workload changes. Detailed results are provided in Table S3 in Multimedia Appendix 4.

### Agreement Between Caregiver and Staff Ratings

For 5 matched items, ratings from caregivers and staff were compared using Mann-Whitney *U* tests with FDR correction (Figure 4). Four of 5 comparisons showed no significant differences: children’s stress and anxiety (*P*=.92), children’s adaptation (*P*=.36), caregivers’ stress and anxiety (*P*=.36), and communication with caregivers (*P*=.61). Only communication with children showed a significant difference (*P*=.03), with staff rating perceived improvement higher than caregivers (mean 3.94, SD 0.72 vs mean 3.56, SD 0.65).

### Qualitative Findings

**Figure 5.**
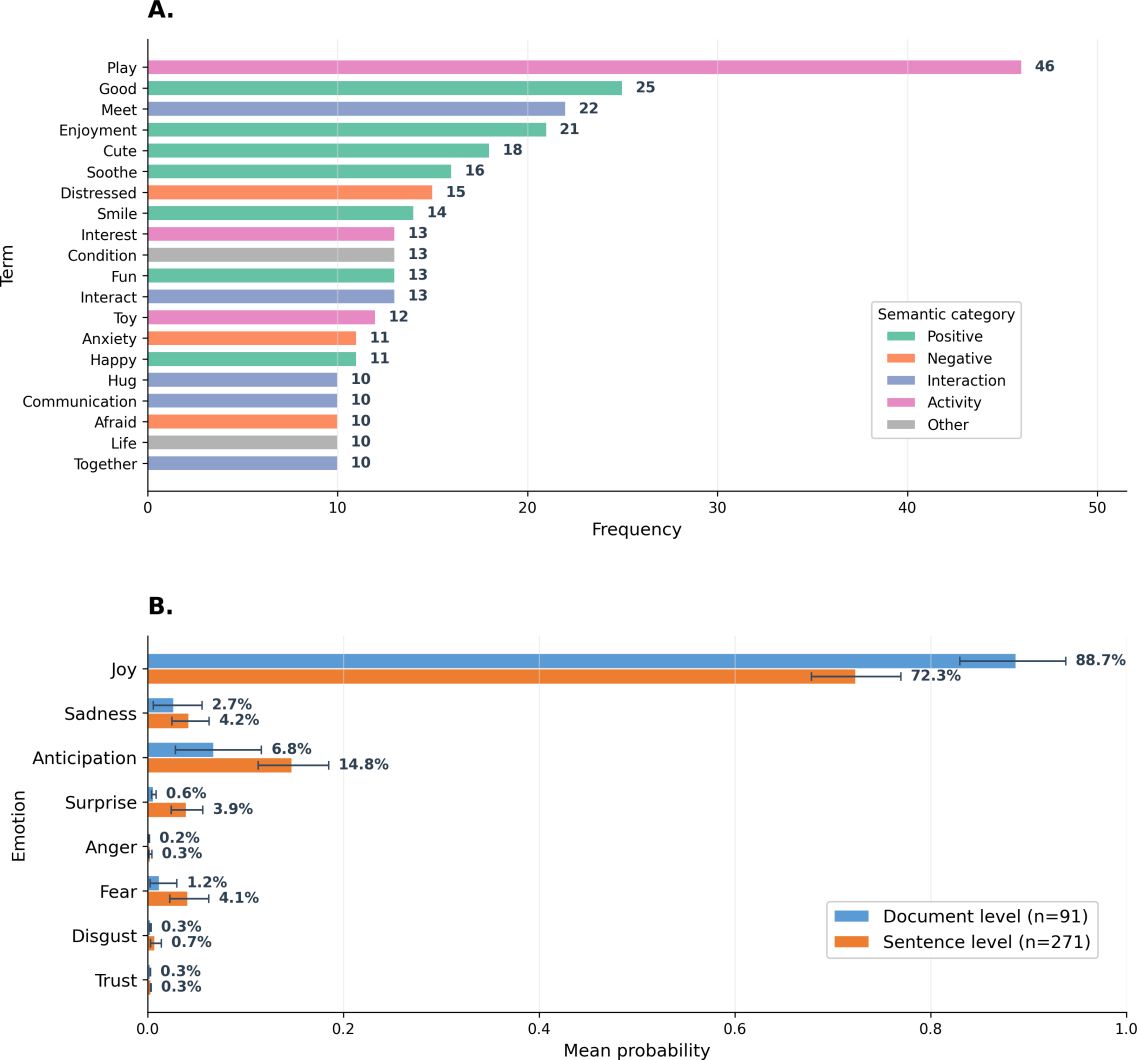
Qualitative analysis of free-text responses (n=91): (A) top 20 high-frequency terms in the Japanese free-text responses, shown with their English translations (translation dictionary provided in Table S2 in Multimedia Appendix 3) and (B) emotion distribution comparing document-level (n=91) and sentence-level (n=271) classification using the LUKE-Japanese-large model. The bars represent mean emotion probabilities with 95% CIs (bootstrap: 5000 iterations).

Analysis of 91 free-text responses revealed predominantly positive experiences (Figure 5). The most frequent terms included “play” (n=46), “good” (n=25), “meet” (n=22), “enjoyment” (n=21), and “cute” (n=18), reflecting themes of play, positive evaluation, and emotional engagement. Emotion classification using the LUKE-Japanese-large model classified “joy” as the predominant emotion at both the document level (mean probability 88.7%, 95% CI 86.8%-90.6%) and sentence level (mean probability 72.3%, 95% CI 69.1%-75.5%). At the document level, most responses (82/91, 90.1%) were classified as joy dominant. Other emotions detected at lower probabilities included anticipation (6.8% at the document level and 14.8% at the sentence level) and fear (1.2% at the document level and 4.1% at the sentence level). The predominance of joy and the prominence of play and positive evaluation themes in the qualitative responses are consistent with the quantitative findings of perceived improvement in children’s enjoyment and adaptation, and the 2 analyses thus reinforce each other.

## Discussion

### Principal Results

This study examined the perceived impact of LOVOT, a companion robot, on hospitalized children, their caregivers, and health care staff. Both caregivers and staff reported consistent perceived improvements across nearly all assessed outcomes, with mean ratings ranging from 3.28 to 4.62 on a 5-point scale. The largest perceived improvements were observed for children’s enjoyment and overall well-being. However, because these effect sizes were calculated from 1-sample tests comparing retrospective ratings against a fixed neutral baseline (3=“no change”) rather than a control group, they are inherently larger than between-group effect sizes from controlled designs and should not be directly compared to those from randomized controlled trials. Importantly, no detectable increase in staff workload was observed, supporting implementation feasibility, although the small staff sample (n=32) limits power to detect small to medium workload changes. Accordingly, the nonsignificant workload result (*P*=.37) should be interpreted as the absence of a detectable increase rather than as evidence of no change and warrants confirmation in adequately powered studies. The convergence of findings across independent observer perspectives and predominantly positive qualitative responses strengthens confidence in these observations.

### Comparison With Prior Work

The positive effects reported are consistent with the broader literature on social robots in health care settings. LOVOT, a companion robot designed for emotional engagement rather than task performance, shares characteristics with other social robots studied in pediatric care, such as Paro [14] and humanoid robots [15-17]. The 1-sample effect sizes in our study appear comparable to or larger than those previously reported [19]; however, direct comparison is limited by fundamental methodological differences as our 1-sample design yielded inherently larger effect sizes than the between-group designs used in most prior studies. Our findings also align with research on animal-assisted therapy, which has demonstrated reductions in anxiety and distress in hospitalized children [8,9]. However, animal-assisted therapy faces practical barriers in busy pediatric wards, including infection control concerns, the need for trained animal handlers, and inconsistent availability due to animal welfare requirements [10,11]. LOVOT offers practical advantages as a robotic alternative: it can be disinfected regularly, operates autonomously without requiring a handler, and provides 24-hour availability without rest periods. Its design, emphasizing passive, tactile interaction through warmth and softness rather than verbal communication, may be particularly suited to pediatric settings where children of varying ages interact freely.

### Possible Mechanisms

Several mechanisms may account for these effects. Physical contact with soft, warm objects reduces stress through parasympathetic activation and oxytocin release [22,23]. The presence of tactile-related terms such as “hug” and “touch” in free-text responses supports the importance of physical contact. LOVOT may also function as a positive distraction, redirecting attention from medical concerns to enjoyable interaction [24,25]. Furthermore, LOVOT appears to serve as a social catalyst; staff reported improved communication with both children and caregivers, and “meet” was among the most frequent terms in qualitative responses. Some caregivers described attachment-like relationships developing, with temporal emotional transitions (initial fear→subsequent joy) suggesting LOVOT may facilitate adaptation to the hospital environment.

### Implementation Feasibility

An important practical finding is that no detectable increase in staff workload was observed despite positive effects across other domains, addressing concerns about introducing new interventions in resource-constrained settings. This may be attributed to LOVOT’s autonomous features, including automatic return to its charging station, which minimizes the need for staff intervention. The minimal training required and LOVOT’s self-directed operation make it a low-burden intervention, distinguishing it from other therapeutic interventions that require dedicated staff time. Subgroup analyses revealed consistent effects across age, hospital stay duration, sex, and playroom use frequency, suggesting broad applicability. The substantial improvement in ward atmosphere indicates benefits extending beyond individual interactions. These findings support LOVOT as a feasible addition to pediatric care, complementing rather than replacing existing human-centered psychosocial support. For pediatric wards considering a similar deployment, our experience suggests that a shared playroom placement, a simple infection control routine (regular disinfection and laundering of removable clothing), brief staff orientation, and reliance on the device’s autonomous operation are sufficient for day-to-day use. These observations are offered as preliminary practical guidance and require confirmation in controlled, multicenter evaluations.

### Limitations

This study has several limitations. First, it relied on retrospective self-report measures of perceived change at a single postintroduction time point rather than prospective pretest-posttest assessments using validated clinical scales. No baseline data were collected prior to LOVOT introduction. Retrospective ratings are subject to recall bias, social desirability bias, and response shift bias (where participants’ internal standards for evaluating change may shift over time), potentially inflating perceived improvements. Unlike a prospective pretest-posttest design, which captures contemporaneous baseline and follow-up measurements, our single–time point retrospective approach cannot separate perceived improvement attributable to LOVOT from natural recovery or adaptation occurring over the course of hospitalization. Thus, the reported improvements reflect caregivers’ and staff’s subjective perceptions rather than objective clinical changes. The observational design without a control group further limits causal inference as improvements may reflect natural adaptation or placebo effects, the stimulating playroom environment itself, or the novelty of interacting with a robot. Our design cannot disentangle these influences from any LOVOT-specific contribution. Additionally, no primary outcome was prespecified. Voluntary participation may have introduced selection bias at multiple levels: caregivers who had positive experiences with LOVOT may have been more motivated to complete the questionnaire, and because questionnaires were available in the playroom, respondents were inherently those who used the playroom—creating a compound engagement bias favoring families with greater LOVOT exposure. Consistent with this, 62.9% (66/105) of respondents reported using the playroom at least once daily, which may overrepresent high-exposure families. The estimated response rate was 18.4% (115/625); however, the denominator was itself estimated based on the assumption that 25% of admitted children used the playroom, and the actual number of unique playroom users was not systematically recorded. This low and uncertain response rate means that findings may not represent the experiences of all playroom users and likely overestimate the perceived benefits of LOVOT. The staff sample (n=32) was relatively small, and the single-center design in Japan limits generalizability to other cultural contexts. Notably, Japanese culture has historically demonstrated a high affinity for robots, which may have positively influenced not only acceptance and willingness to interact but also the magnitude of perceived emotional benefits; whether similar effects would be observed in cultures with different attitudes toward robots remains to be determined. In regions where attitudes toward social robots are more skeptical, both acceptance and the magnitude of perceived psychosocial benefit may be attenuated; cross-cultural, multicenter replication is therefore needed before these findings can be generalized internationally. The study-specific questionnaires showed acceptable to good internal consistency (Cronbach α=0.67-0.84); however, formal psychometric validation including test-retest reliability and construct validity was not conducted. Without established construct validity, the extent to which items accurately capture the intended constructs remains uncertain, and the questionnaire items were designed to assess distinct facets of LOVOT’s impact rather than a single unidimensional construct. The emotion classification model (LUKE-Japanese-large) was fine-tuned on general Japanese text rather than medical or pediatric contexts, and its validity for analyzing health care–related free-text responses has not been established. Because the model was trained on social media–style text, domain shift relative to caregivers’ and staff’s clinical free-text responses may bias the estimated emotion probabilities; we therefore treat the emotion classification results as exploratory and corroborative rather than confirmatory. The convergence between model-based classification and the model-independent high-frequency term analysis provides partial triangulation. We could not link the perceived outcomes to objective measures of interaction intensity: the device’s internal interaction logs (eg, touch and hold frequency) were not available for research export under our deployment configuration, and no separate behavioral observation log was maintained. Integrating objective sensor- and behavior-derived metrics (touch frequency, hold duration, proximity, and interaction duration) is an important direction for future controlled studies and would strengthen the link between interaction and perceived benefit. Finally, the observation period (approximately 11 months) may not fully capture novelty effects or long-term sustainability, although subgroup analyses showed consistent effects among children hospitalized for longer periods (>1 week), suggesting that benefits may persist beyond initial novelty. Future studies should use randomized controlled designs with objective outcome measures, include longitudinal follow-up, and involve multiple centers across diverse cultural contexts.

### Conclusions

This study provides preliminary evidence that LOVOT may positively affect hospitalized children, caregivers, and staff across multiple psychosocial outcomes. The convergence of findings across multiple stakeholder perspectives and analytical methods strengthens confidence in these observations, and positive effects were achieved without a detectable increase in staff workload. While the observational design and low response rate limit causal conclusions and generalizability, these findings justify further investigation through controlled trials with prospective outcome measures. Companion robots represent a promising adjunct to psychosocial care in pediatric settings.

## Supplementary material

10.2196/93897 Multimedia Appendix 1 Supplementary methods: detailed technical specifications of the LOVOT companion robot and the detailed statistical analysis methods.

10.2196/93897 Multimedia Appendix 2 Questionnaire items.

10.2196/93897 Multimedia Appendix 3 Text analysis materials.

10.2196/93897 Multimedia Appendix 4 Statistical results.

10.2196/93897 Checklist 1 STROBE checklist.

## Abbreviations

FDR: false discovery rate
STROBE: Strengthening the Reporting of Observational Studies in Epidemiology

## References

[1] Coyne I (2006). Children’s experiences of hospitalization. J Child Health Care.

[2] Franck LS, Wray J, Gay C, Dearmun AK, Lee K, Cooper BA (2015). Predictors of parent post-traumatic stress symptoms after child hospitalization on general pediatric wards: a prospective cohort study. Int J Nurs Stud.

[3] Delvecchio E, Salcuni S, Lis A, Germani A, Di Riso D (2019). Hospitalized children: anxiety, coping strategies, and pretend play. Front Public Health.

[4] He HG, Zhu LX, Chan WC (2015). A mixed-method study of effects of a therapeutic play intervention for children on parental anxiety and parents’ perceptions of the intervention. J Adv Nurs.

[5] Li WH, Chung JO, Ho KY, Kwok BM (2016). Play interventions to reduce anxiety and negative emotions in hospitalized children. BMC Pediatr.

[6] He HG, Zhu L, Chan SW (2015). Therapeutic play intervention on children’s perioperative anxiety, negative emotional manifestation and postoperative pain: a randomized controlled trial. J Adv Nurs.

[7] Signorelli C, Robertson EG, Valentin C, Alchin JE, Treadgold C (2023). A review of creative play interventions to improve children’s hospital experience and wellbeing. Hosp Pediatr.

[8] Crossman MK (2017). Effects of interactions with animals on human psychological distress. J Clin Psychol.

[9] Fine AH, Beck AM, Ng Z (2019). The state of animal-assisted interventions: addressing the contemporary issues that will shape the future. Int J Environ Res Public Health.

[10] Lefebvre SL, Golab GC, Writing Panel of Working Group (2008). Guidelines for animal-assisted interventions in health care facilities. Am J Infect Control.

[11] Brodie SJ, Biley FC, Shewring M (2002). An exploration of the potential risks associated with using pet therapy in healthcare settings. J Clin Nurs.

[12] Logan DE, Breazeal C, Goodwin MS (2019). Social robots for hospitalized children. Pediatrics.

[13] Moerman CJ, van der Heide L, Heerink M (2019). Social robots to support children’s well-being under medical treatment: a systematic state-of-the-art review. J Child Health Care.

[14] Geva N, Uzefovsky F, Levy-Tzedek S (2020). Touching the social robot PARO reduces pain perception and salivary oxytocin levels. Sci Rep.

[15] Beran T, Pearson JR, Lashewicz B, Baggott S (2020). Perspectives of child life specialists after many years of working with a humanoid robot in a pediatric hospital: narrative design. J Med Internet Res.

[16] Beran TN, Pearson JR, Lashewicz B (2021). Implementation of a humanoid robot as an innovative approach to child life interventions in a children’s hospital: lofty goal or tangible reality?. Front Psychol.

[17] Littler BK, Alessa T, Dimitri P, Smith C, de Witte L (2021). Reducing negative emotions in children using social robots: systematic review. Arch Dis Child.

[18] LOVOT.

[19] Dawe J, Sutherland C, Barco A, Broadbent E (2019). Can social robots help children in healthcare contexts? A scoping review. BMJ Paediatr Open.

[20] Iwai A LOVOT pediatric study - analysis code. GitHub.

[21] Sawilowsky SS (2009). New effect size rules of thumb. J Mod Appl Stat Methods.

[22] Field T (2010). Touch for socioemotional and physical well-being: a review. Dev Rev.

[23] Uvnäs-Moberg K, Handlin L, Petersson M (2015). Self-soothing behaviors with particular reference to oxytocin release induced by non-noxious sensory stimulation. Front Psychol.

[24] Koller D, Goldman RD (2012). Distraction techniques for children undergoing procedures: a critical review of pediatric research. J Pediatr Nurs.

[25] Ali S, Manaloor R, Ma K (2021). A randomized trial of robot-based distraction to reduce children’s distress and pain during intravenous insertion in the emergency department. CJEM.

