# Supplementary material for: Impact of a Social Robot on Hospitalized Children, Caregivers, and Health Care Staff: Exploratory Observational Study
Source: JMIR Pediatr Parent. 2026 Jul 10;9:e93897. doi: 10.2196/93897 (PMC13352968; doi:10.2196/93897)
Supplement: Multimedia Appendix 1 [file pediatrics-v9-e93897-s001.pdf]

Multimedia Appendix 1. Supplementary Methods

Article Title: Impact of a Social Robot on Hospitalized Children, Caregivers, and Health Care Staff: Exploratory Observational Study

Journal: JMIR Pediatrics and Parenting

LOVOT Technical Specifications

LOVOT 2.0 (Groove X, Inc., Tokyo, Japan) is a companion robot designed for emotional engagement through non-verbal communication. The detailed technical specifications are as follows:

Physical Characteristics

- **Dimensions:** 280 × 450 × 300 mm (width × height × depth)
- **Weight:** Approximately 4.3 kg (excluding clothing)

Computing and Sensors

- **Processor:** Dual processor system (x86 CPU and ARM CPU)
- **Visual sensors:** Dual LCD eyes, hemispherical camera, thermal camera, depth camera for environmental sensing
- **Other sensors:** Multiple touch sensors distributed across the body surface

Communication

- **Wireless:** Bluetooth, Wi-Fi, mobile data connectivity

Power

- **Battery:** Li-ion battery (89 Wh capacity)
- **Operating time:** Approximately 30–45 minutes per charging cycle
- **Charging time:** Approximately 15–30 minutes
- **Charging behavior:** LOVOT automatically returns to its charging station (nest) when battery is low

Design Features Relevant to Pediatric Use

- **Body temperature:** Maintained at approximately 37°C to simulate warmth of a living creature
- **Tactile exterior:** Soft, plush covering that encourages physical contact
- **Appearance:** Designed with large eyes and rounded features to evoke nurturing responses
- **Clothing:** Removable and washable garments for infection control

Detailed Statistical Analysis Methods

Software and Packages

All statistical analyses were conducted using R version 4.5.1 (R Foundation for Statistical Computing, Vienna, Austria). The following R packages were used:

- **Data manipulation:** tidyverse (dplyr, tidyr, readr, ggplot2)
- **Statistical analysis:** effsize (effect size calculation), pwr (power analysis)
- **Table creation:** gtsummary (summary tables), gt (table formatting), flextable (Word output)
- **Visualization:** patchwork (multi-panel figures), RColorBrewer (color palettes)

Python version 3.13.7 was used for qualitative text analysis with the following packages:

- **Data processing:** pandas (≥1.3.0), numpy (≥1.20.0)
- **Visualization:** matplotlib (≥3.4.0), wordcloud (≥1.8.0)
- **Japanese NLP:** fugashi (≥1.1.0), unidic-lite (≥1.0.8)
- **Emotion classification:** transformers (≥4.20.0), torch (≥1.10.0)
- **Statistical analysis:** scikit-learn (≥1.0.0), scipy (≥1.7.0)

Complete package version information is available in the requirements.txt file in the GitHub repository.

Quantitative Analysis: Detailed Methods

Study Design Considerations

This was an **exploratory analysis without pre-specified primary outcomes**. All questionnaire items were treated equally with appropriate multiple testing correction applied. The exploratory nature of the analysis should be considered when interpreting results, as hypothesis generation rather than hypothesis testing was the primary goal.

Primary Analysis: One-Sample Tests

Rationale: The study aimed to assess whether LOVOT introduction was associated with changes from baseline (defined as "no change" = 3 on the Likert scale). One-sample tests comparing observed ratings against this baseline value of 3 were therefore appropriate.

Normality Assessment:

- The Shapiro-Wilk test was performed for all variables
- Variables with $P \geq .05$ were considered normally distributed
- Variables with $P < .05$ were considered non-normally distributed

Test Selection:

- **For normally distributed data ($P \geq .05$):** One-sample t-test
    - Null hypothesis: $\mu = 3$ (no change)
    - Alternative hypothesis: $\mu \neq 3$ (change from baseline)
    - Two-tailed test with $\alpha = 0.05$ (before FDR correction)
- **For non-normally distributed data ($P < .05$):** Wilcoxon signed-rank test
    - Null hypothesis: median = 3 (no change)
    - Alternative hypothesis: median ≠ 3 (change from baseline)

  - Two-tailed test with $\alpha = 0.05$ (before FDR correction)

Test Implementation:

- For t-tests: Used R base `t.test()` function
- For Wilcoxon tests: Used R base `wilcox.test()` function with `exact = FALSE` (due to ties) and `conf.int = TRUE` to obtain Hodges-Lehmann estimator confidence intervals

Multiple Testing Correction

Rationale: Testing multiple outcomes increases the risk of false positives (Type I error). Multiple testing correction was applied to control the family-wise error rate.

Method: Benjamini-Hochberg Procedure

- Controls the False Discovery Rate (FDR) rather than family-wise error rate
- More powerful than Bonferroni correction while still providing appropriate Type I error control
- Implemented using R's `p.adjust()` function with `method = "BH"`

Application:

- **Caregiver ratings:** FDR correction applied across 10 items (Q3-Q9, Q11-Q13)
- **Staff ratings:** FDR correction applied across 12 items (Panel A: 7 items, Panel B: 5 items)
- **Subgroup analyses:** FDR correction applied separately within each subgroup across 7 child outcome items (Q3-Q9)
- **Caregiver vs. Staff comparison:** FDR correction applied across 5 matched items

Significance Threshold:

- FDR-corrected P value <.05 was considered statistically significant
- Both raw and FDR-corrected P values are reported in Multimedia Appendix 4

Effect Size Calculation

Cohen d for One-Sample Tests:

- Calculated as: $d = (M - \mu_0) / SD$
  - M = sample mean
  - μ_0 = baseline value (3)
  - SD = sample standard deviation
- Implemented using the `effsize` package `cohen.d()` function

Interpretation Thresholds:

- Negligible: $|d| < 0.2$
- Small: $0.2 \le |d| < 0.5$
- Medium: $0.5 \le |d| < 0.8$
- Large: $0.8 \le |d| < 1.2$
- Very large: $1.2 \le |d| < 2.0$
- Huge: $|d| \ge 2.0$

Rationale for Reporting Effect Sizes: Effect sizes provide information about the magnitude and practical significance of effects, complementing statistical significance tests. With the sample sizes in this study (n=110 caregivers, n=32 staff), even small effects may reach statistical significance, making effect size interpretation particularly important.

Confidence Intervals

For t-tests:

- 95% parametric confidence intervals calculated using t-distribution
- Confidence intervals represent the range of plausible values for the true population mean

For Wilcoxon tests:

- 95% confidence intervals calculated using the Hodges-Lehmann estimator
- The Hodges-Lehmann estimator is a robust measure of central tendency appropriate for non-parametric tests
- Confidence intervals obtained using `wilcox.test(..., conf.int = TRUE)`

Visualization:

- Forest plots display mean differences from baseline (3) with 95% confidence intervals
- For visual consistency, t-test confidence intervals are shown in all forest plots regardless of which test was used for significance testing

Subgroup Analysis

Stratification Variables:

1. **Age group:**
   - Younger children (≤5 years) vs. Older children (≥6 years)
   - Rationale: Developmental differences may influence LOVOT interaction patterns
2. **Hospital stay duration:**
   - Short-term (≤1 week) vs. Longer-term (>1 week)
   - Rationale: Length of exposure to LOVOT may influence effects
3. **Sex:**
   - Female vs. Male
   - Rationale: Exploratory assessment of sex differences
4. **Playroom use frequency:**
   - High (at least once per day) vs. Low-medium (less than once per day)
   - Rationale: Frequency of LOVOT exposure may influence effects

Within-Subgroup Analysis:

- Same one-sample tests as primary analysis (baseline = 3)
- Normality assessed separately within each subgroup

- T-test or Wilcoxon test applied as appropriate
- FDR correction applied separately within each subgroup across 7 child outcomes (Q3-Q9)

Between-Subgroup Comparison:

- **Test used:** Mann-Whitney U test (also called Wilcoxon rank-sum test)
- **Rationale:** Non-parametric test appropriate for comparing independent groups with small sample sizes and potential non-normal distributions
- **Null hypothesis:** No difference in distributions between two subgroups
- **FDR correction:** Applied across 7 child outcomes within each stratification variable
- **Implementation:** R base `wilcox.test()` function with paired = FALSE

Important Note: Subgroup analyses were not pre-specified and should be considered exploratory. They assess the consistency of effects across patient characteristics rather than testing specific hypotheses about differential effects.

Caregiver vs. Staff Comparison

Matched Items (n=5):

1. Children's stress/anxiety (Q7 vs. child_stress)
2. Children's adaptation (Q9 vs. child_adaptation)
3. Caregivers' stress/anxiety (Q13 vs. caregiver_stress)
4. Communication with children (Q5 vs. child_comm)
5. Communication with caregivers (Q12 vs. caregiver_comm)

Test Used: Mann-Whitney U test

- **Rationale:** Comparing independent samples (caregivers vs. staff) with non-normal distributions
- **Null hypothesis:** No difference between caregiver and staff ratings
- **FDR correction:** Applied across 5 comparisons
- **Implementation:** R base `wilcox.test()` with paired = FALSE

Interpretation: Agreement between independent observer perspectives (caregivers and staff) supports the objectivity of observed effects. Significant differences may reflect different observational vantage points rather than contradictory assessments.

Qualitative Analysis: Detailed Methods

Overview

Free-text responses (n=91) in Japanese were analyzed using automated text analysis methods. **Important note:** This represents an exploratory automated analysis rather than traditional qualitative research with manual coding schemes, thematic analysis, or grounded theory approaches. Results should be interpreted as computational indicators of overall sentiment patterns rather than in-depth qualitative insights.

Text Preprocessing

1. Morphological Analysis:

- **Tool:** MeCab with UniDic dictionary via fugashi library (Python)
- **Purpose:** Segment Japanese text into morphemes (smallest meaningful units)
- **Process:**
  - Each response tokenized into individual morphemes
  - Part-of-speech (POS) tags assigned to each token
  - Lemma (dictionary form) extracted for normalization

2. Token Normalization:

- **Reading-based normalization:** Tokens converted to hiragana readings to ensure consistency (e.g., kanji variants normalized to common reading)
- **Lemma mapping:** Custom dictionary applied to normalize common variants (e.g., "呉れる" → "くれる", "下さる" → "くださる")
- **Rationale:** Japanese text uses multiple writing systems (kanji, hiragana, katakana) for the same concepts; normalization ensures accurate frequency counts

3. Part-of-Speech Filtering:

- **Retained POS tags:** Nouns (名詞), Adjectives (形容詞), Verbs (動詞)
- **Excluded POS tags:** Particles, auxiliary verbs, punctuation
- **Rationale:** Content words (nouns, adjectives, verbs) carry semantic meaning relevant to thematic analysis

4. Katakana Phrase Joining:

- Consecutive katakana tokens joined to form multi-word phrases
- **Example:** "ロ" + "ボット" → "ロボット" (robot)
- **Rationale:** Katakana is often used for foreign loanwords; joining prevents fragmentation of meaningful terms

5. Stopword Removal:

- **Domain-specific stopword list:** 91 terms manually curated (Multimedia Appendix 3, Table S1)
- **Categories of stopwords:**
  - Common function words (e.g., "する" = to do, "ある" = to exist)
  - Generic temporal/spatial terms (e.g., "時" = time, "所" = place)
  - Generic descriptors (e.g., "事" = thing, "方" = person/way)
  - Medical context-specific common terms (e.g., "病院" = hospital)
- **Rationale:** Stopwords are high-frequency but low-information terms that would dominate visualizations without providing meaningful insights

6. Mixed Language Token Cleaning:

- Tokens containing both Japanese and English characters processed to remove English portions
- **Example:** "プレイルーム(playroom)" → "プレイルーム"
- **Rationale:** Parenthetical English translations added by respondents are redundant for Japanese text analysis

7. Document Frequency Filtering:

- Tokens appearing in less than 3 documents excluded

- **Rationale:** Very rare terms (hapax legomena, idiosyncratic expressions) may be noise rather than signal; minimum document frequency threshold improves robustness

8. Minimum Token Length:

- Tokens with <2 characters excluded
- **Rationale:** Single-character tokens are typically non-informative

Word Frequency Analysis

1. Frequency Calculation:

- After preprocessing, term frequency calculated across all documents
- Total corpus vocabulary: [N unique terms after filtering]

2. Term Frequency Visualization:

- **Tool:** Python matplotlib library
- **Minimum frequency:** Terms appearing ≥3 times in corpus
- **Visualization:** Horizontal bar chart displaying the top 20 most frequent terms
- **Color coding:** Bars colored by semantic category to facilitate interpretation
- **Two versions created:**
  - Japanese term frequency chart (original terms)
  - English term frequency chart (translated terms using custom dictionary)

3. Term Translation:

- Custom Japanese-English translation dictionary developed (Multimedia Appendix 3, Table S2)
- **Translation process:**
  - High-frequency terms manually translated by bilingual researcher
  - Context-appropriate translations chosen (e.g., "触る" = "touch/interact" rather than literal "touch")
  - Medical/pediatric context considered for domain-specific terms

Emotion Classification

Model:

- **Name:** LUKE-Japanese-large-sentiment-analysis-wrime
- **Source:** Mizuiro-sakura/luke-japanese-large-sentiment-analysis-wrime (Hugging Face)
- **Architecture:** LUKE (Language Understanding with Knowledge-based Embeddings) fine-tuned for Japanese emotion classification
- **Training data:** WRIME (Wakamiya's Readers' Impression of Emotions) dataset
  - Japanese blog posts with emotion annotations
  - **Important limitation:** Model trained on general text, not pediatric medical contexts

Emotion Categories (8 emotions):

1. Joy (喜び)
2. Sadness (悲しみ)
3. Anticipation (期待)

4. Surprise (驚き)
5. Anger (怒り)
6. Fear (恐れ)
7. Disgust (嫌悪)
8. Trust (信頼)

Classification Levels:

1. **Document-Level Classification:**
    - Each free-text response classified as a whole
    - Produces single emotion distribution per respondent
    - **Aggregation:** Average emotion proportions across all n=91 responses
2. **Sentence-Level Classification:**
    - Each response segmented into sentences using Japanese sentence-ending patterns (。！？)
    - Each sentence classified independently
    - **Aggregation:** Average emotion proportions across all sentences from all responses
    - **Rationale:** Provides finer-grained view; checks consistency with document-level analysis

Inference Process:

- Tokenization using LUKE tokenizer (MLukeTokenizer)
- Maximum sequence length: 512 tokens (LUKE limitation)
- For longer responses, truncation applied
- Softmax probabilities computed for 8 emotion classes
- Emotion with highest probability assigned to each text unit

Bootstrap Confidence Intervals

Purpose: Quantify uncertainty in emotion distribution estimates

Method:

- **Resampling:** 5,000 bootstrap iterations
- **Process:**
    1. Random sampling with replacement from n=91 responses
    2. Calculate emotion distribution for bootstrap sample
    3. Repeat 5,000 times
    4. Obtain distribution of emotion proportions across bootstrap samples
- **Confidence Interval Calculation:**
    - Percentile method: 2.5th and 97.5th percentiles of bootstrap distribution
    - Produces 95% confidence interval for each emotion category

Interpretation:

- Narrow confidence intervals indicate stable, reliable estimates
- Wide confidence intervals indicate higher uncertainty due to sample variability

Model Limitations and Interpretation Guidance

Key Limitations:

1. **General vs. Domain-Specific Model:**
   - LUKE model trained on general Japanese text (blogs), not pediatric medical contexts
   - Emotion expressions in hospital settings may differ from training data
   - Model may not capture medical context-specific sentiment nuances
2. **Automated vs. Manual Analysis:**
   - No manual validation of emotion classifications
   - No inter-rater reliability assessment (as would be standard in qualitative research)
   - Cannot capture subtle contextual meanings or cultural nuances
3. **Emotion Categories:**
   - 8 pre-defined categories may not fully represent emotional experiences in hospital settings
   - Respondents' actual emotional states may be more complex or nuanced than model categories
4. **Language-Specific Challenges:**
   - Japanese emotion expressions may not map directly to Western emotion taxonomies
   - Cultural differences in emotional expression and reporting

Appropriate Interpretation:

- Results represent **computational indicators of overall sentiment patterns**
- Should be interpreted as **exploratory, descriptive findings**
- **Not validated psychological measures** of emotional states
- Best used to complement quantitative questionnaire data and provide qualitative context
- Convergence with quantitative findings (e.g., high "joy" aligning with high enjoyment ratings) provides triangulation evidence

---

Missing Data Handling

Caregiver Questionnaire:

- Complete case analysis: Only respondents with non-missing values for each item included in that item's analysis
- Sample size (n) reported for each analysis reflects available data
- Missing data patterns examined; no systematic patterns identified

Staff Questionnaire:

- Complete case analysis applied

- Small sample size (n=32) limited options for imputation methods

Rationale:

- Missing data proportion was low (<5% for most items)
- Complete case analysis is appropriate when missing completely at random (MCAR)
- Sensitivity analyses with imputation were not performed given low missingness

---

Software Code Availability

All analysis code is publicly available under the MIT License at: https://github.com/iwai-research/lovot-analysis

The repository includes:

- R scripts for all quantitative analyses (Tables, Figures 1-4)
- Python scripts for qualitative analysis (Figure 5)
- requirements.txt for Python package versions
- Complete documentation and README files
- Data codebook and variable definitions

Reproducibility: Complete reproducibility is possible with the provided code and data (subject to institutional review board approval for data access).

Summary of Analytical Decisions and Rationale

Decision	Rationale
Exploratory analysis (no primary outcome)	Study was observational and hypothesis-generating rather than hypothesis-testing
One-sample tests vs. baseline=3	Assessed change from "no change" baseline on Likert scale
Shapiro-Wilk for normality	Standard test; appropriate for sample sizes n=32-110
T-test vs. Wilcoxon based on normality	Maximizes statistical power while maintaining appropriate assumptions
Benjamini-Hochberg FDR correction	More powerful than Bonferroni; appropriate for exploratory analysis
Cohen d effect sizes	Provides magnitude information complementing significance tests
Mann-Whitney U for subgroups	Non-parametric; appropriate for independent samples with small n
Automated text analysis	Feasible for n=91 responses; provides quantitative complement to qualitative themes

Decision	Rationale
LUKE emotion model	Best available Japanese emotion classification model; pre-trained on large corpus
Bootstrap CIs (5,000 iterations)	Standard approach for quantifying uncertainty in distribution estimates

Adherence to Reporting Guidelines

This study followed the STROBE (Strengthening the Reporting of Observational Studies in Epidemiology) guidelines for reporting observational studies where applicable.

Statistical analyses were conducted and reported following recommendations from:

- American Statistical Association (ASA) Statement on P-Values and Statistical Significance
- SAMPL Guidelines (Statistical Analyses and Methods in the Published Literature)
- Transparent Statistics Guidelines

Key principles applied:

- Effect sizes reported alongside P values
- Multiple testing correction explicitly described
- Exploratory nature of analysis clearly stated
- Confidence intervals provided for all estimates
- Complete methods transparency with code sharing
