# Supplementary material for: Impact of a Social Robot on Hospitalized Children, Caregivers, and Health Care Staff: Exploratory Observational Study
Source: JMIR Pediatr Parent. 2026 Jul 10;9:e93897. doi: 10.2196/93897 (PMC13352968; doi:10.2196/93897)
Supplement: Multimedia Appendix 2 [file pediatrics-v9-e93897-s002.pdf]

Multimedia Appendix 2. Questionnaire Items

Table S1. Caregiver Questionnaire Items

Complete questionnaire administered to caregivers (parents/guardians) of hospitalized children who used the pediatric playroom during the LOVOT deployment period. All outcome items used a 5-point Likert scale to assess changes compared to before LOVOT introduction.

Response Scale

Score	Meaning
1	Much worse than before
2	Slightly worse than before
3	No change
4	Slightly better than before
5	Much better than before

Section A: Child-Related Outcomes (Caregiver Report)

Item No	Variable	English Item	Japanese Item (Original)	Domain
Q3	Q3_smile	Frequency of child's smiles	LOVOTと遊ぶことで、お子さんが笑顔を見せる頻度はどの程度変化しましたか？	Positive emotion
Q4	Q4_peer_play	Time playing with peers	LOVOTと遊ぶことで、お子さんが他の子どもたちと一緒に遊ぶ時間はどのように変化しましたか？	Social interaction
Q5	Q5_staff_comm	Frequency of child's communication with staff	LOVOTがプレイルームにいることで、お子さんが医療スタッフとコミュニケーションを取る頻度はどのように変化しましたか？	Communication
Q6	Q6_physical_activity	Time in physical activity	LOVOTと遊ぶことで、お子さんの身体を動かす時間はどのように変化しましたか？	Activity level
Q7	Q7_stress_anxiety	Child's stress/anxiety	LOVOTと遊ぶことで、お子さんのストレスや不安はど	Negative emotion

Item No	Variable	English Item	Japanese Item (Original)	Domain
			のように変化したと感じましたか？	
Q8	Q8_enjoyment	Enjoyment of hospital life	LOVOTがプレイルームにいることで、お子さんの入院生活における楽しみはどのように変化したと感じましたか？	Overall well-being
Q9	Q9_adaptation	Adaptation to hospital life	LOVOTとの関わりが、お子さんの入院生活への適応にどのように影響したと感じますか？	Overall well-being

Section B: Caregiver-Related Outcomes

Item No	Variable	English Item	Japanese Item (Original)	Domain
Q11	Q11_child_comm	Frequency of communication with child	LOVOTがプレイルームにいることで、あなたがお子さんとコミュニケーションを取る頻度はどのように変化しましたか？	Communication
Q12	Q12_staff_comm_cg	Frequency of communication with staff	LOVOTがプレイルームにいることで、あなたが医療スタッフとコミュニケーションを取る頻度はどのように変化しましたか？	Communication
Q13	Q13_caregiver_stress	Own stress/anxiety	LOVOTと関わることで、あなた自身のストレスや不安はどのように変化しましたか？	Negative emotion

Additional Demographic Items

The questionnaire also included demographic items (Q1-Q2, Q10, Q14-Q17) to collect information about child age and sex, hospitalization duration, playroom use frequency, LOVOT contact status, and caregiver age and sex. These are reported in Table 1 of the main manuscript.

Notes

1. Only caregivers who reported having LOVOT contact (n=110) were included in outcome analyses
2. Statistical results are provided in Multimedia Appendix 4, Table S1
3. Self-administered paper survey completed at end of study period

4. English items are back-translations for international readers; the original survey was in Japanese
5. Face validity was confirmed by pediatric health care professionals

Total outcome items: 10 (Q3-Q9, Q11-Q13)

Table S2. Health Care Staff Questionnaire Items

Complete questionnaire administered to health care staff (physicians, nurses, childcare workers) working in the pediatric ward during the LOVOT deployment period. All items used a 5-point Likert scale to assess changes compared to before LOVOT introduction.

Response Scale

Same 5-point Likert scale as the caregiver questionnaire (see Table S1).

Note: For Item 12 (workload), "worse" indicates increased workload and "better" indicates decreased workload.

Panel A: Impact on Children and Caregivers (Staff Perspective)

Item No	Variable	English Item	Japanese Item (Original)	Domain
1	child_stress	Children's stress/anxiety	LOVOTと遊ぶことで、入院中の子どもたちのストレスや不安はどのように変化したと感じましたか？	Child emotional state
2	child_adaptation	Children's adaptation to hospital life	LOVOTとの関わりが、入院中の子どもたちの入院生活への適応にどのように影響したと感じましたか？	Child well-being
3	child_impact_overall	Overall impact on children	総合的に判断し、LOVOTが入院中の子どもたちへ与えた影響についてどのように感じましたか？	Child overall
4	caregiver_stress	Caregivers' stress/anxiety	LOVOTと関わることで、付き添いの保護者のストレスや不安はどのように変化したと感じましたか？	Caregiver emotional state
5	caregiver_impact_overall	Overall impact on caregivers	総合的に判断して、LOVOTが付き添いの保護者へ与えた影響について	Caregiver overall

Item No	Variable	English Item	Japanese Item (Original)	Domain
			どのように感じましたか？	
6	child_comm	Communication with children	LOVOTを利用することで、あなたが入院中の患児とコミュニケーションをとる機会はどのように変化したと感じましたか？	Communication
7	caregiver_comm	Communication with caregivers	LOVOTを利用することで、あなたが付き添いの保護者とコミュニケーションをとる機会はどのように変化したと感じましたか？	Communication

Panel B: Impact on Staff and Ward Environment

Item No	Variable	English Item	Japanese Item (Original)	Domain
8	staff_own_stress	Own stress/anxiety	LOVOTと触れ合うことで、あなたのストレスや不安はどのように変化したと感じましたか？	Staff well-being
9	other_staff_stress	Other staff's stress/anxiety	LOVOTと触れ合うことで、他の職員のストレスや不安はどのように変化したと感じましたか？	Staff well-being
10	other_staff_comm	Communication with other staff	LOVOTを利用することで、あなたが他の医療者とコミュニケーションをとる機会はどのように変化したと感じましたか？	Communication
11	ward_atmosphere	Overall ward atmosphere	総合的に判断して、LOVOTが職員や病棟全体の雰囲気へ与えた影響についてどのように感じましたか？	Environment
12	workload	Workload increase	LOVOTを導入することであなたの業務への負担は増加しましたか？	Practical impact

Notes

1. All staff responses (n=32) were included in the analysis
2. Statistical results are provided in Multimedia Appendix 4, Table S3

3. Benjamini-Hochberg FDR correction applied across 12 items
4. Self-administered paper survey completed at end of study period
5. English items are back-translations for international readers; the original survey was in Japanese
6. Face validity was confirmed by pediatric health care professionals and hospital administrators

Total outcome items: 12
