# Supplementary material for: Impact of a Social Robot on Hospitalized Children, Caregivers, and Health Care Staff: Exploratory Observational Study
Source: JMIR Pediatr Parent. 2026 Jul 10;9:e93897. doi: 10.2196/93897 (PMC13352968; doi:10.2196/93897)
Supplement: Multimedia Appendix 3 [file pediatrics-v9-e93897-s003.pdf]

Multimedia Appendix 3. Text Analysis Materials

Table S1. Domain-Specific Stopwords Excluded From Text Mining Analysis

Stopwords were categorized into 5 main groups: (1) function words (n=66), including high-frequency verbs (eg, する [to do], いる [to be]), nouns (eg, こと [thing], 場所 [place]), pronouns (eg, これ [this], それ [that]), and temporal expressions (eg, 今日 [today], 普段 [usually]); (2) institution-specific terms (n=2), such as プレイルーム [playroom]; (3) medical terminology (n=6), such as 入院 [hospitalization] and 病棟 [ward]; (4) study-specific terms (n=8), including LOVOT robot names (eg, ちゃちゃまる [Chachamaru], ちょこまる [Chokomaru]) and generic robot terms; and (5) general high-frequency terms (n=9) without substantive emotional content, such as 子ども [child] and 保護者 [caregiver]. All terms were excluded to focus analysis on substantive emotional and experiential content expressed by caregivers.

Category 1: Function Words (n=66)

Verbs (n=45)

Japanese	English Translation	Notes
する	to do	High-frequency auxiliary verb
なる	to become	High-frequency auxiliary verb
ある	to be/exist	Existential verb
いる	to be (animate)	Existential verb for animate objects
できる	can/to be able to	Potential verb
思う	to think	High-frequency mental verb
感じる	to feel	High-frequency mental verb
いただく	to receive (humble)	Humble auxiliary verb
くださる	to give (honorific)	Honorific auxiliary verb
くれる	to give	Giving auxiliary verb
ください	please	Polite request form
ござる	to be (archaic honorific)	Honorific copula
見る	to see/look	High-frequency perception verb
みる	to see/look	Hiragana form
行く	to go	High-frequency movement verb
いく	to go	Hiragana form
来る	to come	High-frequency movement verb

Japanese	English Translation	Notes
くる	to come	Hiragana form
言う	to say	High-frequency speech verb
いう	to say	Hiragana form
取る	to take	High-frequency action verb
とる	to take	Hiragana form
感ずる	to feel	Variant of 感じる
かんずる	to feel	Hiragana form
分かる	to understand	High-frequency cognitive verb
わかる	to understand	Hiragana form
掛ける	to hang/call	Multi-meaning verb
かける	to hang/call	Hiragana form
やる	to do	Colloquial form of する
知る	to know	High-frequency cognitive verb
しる	to know	Hiragana form
過ごす	to spend (time)	High-frequency temporal verb
すごす	to spend (time)	Hiragana form
しまう	to end up (auxiliary)	Resultative auxiliary verb
持つ	to have/hold	High-frequency possessive verb
もつ	to have/hold	Hiragana form
示す	to show/indicate	High-frequency verb
しめす	to show/indicate	Hiragana form
ない	not/no	Negative auxiliary
入る	to enter	High-frequency movement verb
はいる	to enter	Hiragana form
出る	to exit/leave	High-frequency movement verb
でる	to exit/leave	Hiragana form
ます	polite suffix	Polite verb ending
です	to be (polite)	Polite copula

Nouns (n=10)

Japanese	English Translation	Notes
こと	thing/matter	Abstract nominalizer
もの	thing/object	Generic object
ところ	place/point	Generic location
自体	itself/in itself	Abstract noun
じたい	itself/in itself	Hiragana form
場所	place/location	Generic location
ばしょ	place/location	Hiragana form
時間	time	Generic time term
じかん	time	Hiragana form
ため	for/because	Purpose/reason marker

Pronouns (n=3)

Japanese	English Translation	Notes
それ	that	Demonstrative pronoun
これ	this	Demonstrative pronoun
あれ	that (over there)	Demonstrative pronoun

Temporal Expressions (n=7)

Japanese	English Translation	Notes
今日	today	Temporal noun
きょう	today	Hiragana form
以前	before/previously	Temporal adverb
いぜん	before/previously	Hiragana form
今回	this time	Temporal noun
以上	above/more than	Quantitative marker
普段	usually/normally	Temporal adverb

Other Function Words (n=1)

Japanese	English Translation	Notes
よう	way/like	Manner/similarity marker

Category 2: Institution-Specific Terms (n=2)

Japanese	English Translation	Notes
プレイルーム	playroom	Pediatric ward playroom
プレールーム	playroom (variant)	Variant spelling of playroom

Category 3: Medical Terminology (n=6)

Japanese	English Translation	Notes
病棟	ward	Hospital ward
入院	hospitalization	Hospital admission
病院	hospital	Medical institution
病室	hospital room	Patient room
手術	surgery	Surgical procedure
医療	medical care	Healthcare/medical services

Category 4: Study-Specific Terms (n=8)

Japanese	English Translation	Notes
LOVOT	LOVOT	Social robot brand name
ロボット	robot	Generic robot term
ちゃちゃまる	Chachamaru	LOVOT individual name
ちゃちゃ	Chacha	Shortened LOVOT name
ちょこまる	Chokomaru	LOVOT individual name
ちょこ	Choko	Shortened LOVOT name
テリー	Terry	LOVOT individual name
てりー	Terry	Alternative spelling

Category 5: General High-Frequency Terms (n=9)

Japanese	English Translation	Notes
こども	child/children	Alternative spelling
子ども	child/children	Standard spelling
子供	child/children	Alternative kanji form
保護者	caregiver/guardian	Parent or legal guardian
スタッフ	staff	Medical/hospital staff

Japanese	English Translation	Notes
行動	behavior/action	Generic behavior term
こうどう	behavior/action	Hiragana form
バイバイ	bye-bye	Greeting/farewell
バイ	bye	Shortened form

Total: 91 stopwords (Function words: 66; Institution-specific: 2; Medical terminology: 6; Study-specific: 8; General high-frequency: 9)

Note: Some terms appear in multiple forms (kanji, hiragana) to ensure comprehensive filtering during text preprocessing.

Table S2. Japanese-English Translation Dictionary for Text Analysis

Translation dictionary used to generate the English term frequency visualization from Japanese tokens identified after stopword removal and frequency filtering. All translations were verified by bilingual researchers (AI, HY) to ensure semantic equivalence and contextual appropriateness. Terms are listed in order of frequency.

Japanese	English Translation	Frequency	Semantic Category
遊ぶ	play	46	Activity/Engagement
良い	good	25	Positive evaluation
会う	meet	22	Social interaction
楽しみ	enjoyment	21	Positive emotion
可愛い	cute	18	Positive evaluation
癒す	soothe	16	Emotional support
困る	distressed	15	Negative emotion
笑顔	smile	14	Positive expression
興味	interest	13	Engagement
様子	condition	13	Observation
楽しい	fun	13	Positive emotion
触れ合う	interact	13	Social interaction
玩具	toy	12	Object/Play
不安	anxiety	11	Negative emotion
嬉しい	happy	11	Positive emotion
抱っこ	hug	10	Physical interaction

Japanese	English Translation	Frequency	Semantic Category
コミュニケーション	communication	10	Social interaction
怖い	afraid	10	Negative emotion
生活	life	10	Daily experience
一緒	together	10	Social connection
話し掛ける	talk to	10	Communication
触る	touch	9	Physical interaction
頑張る	make effort	9	Effort/Engagement
動く	move	9	Action/Behavior
楽しむ	enjoy	9	Positive emotion
凄い	wonderful	9	Positive evaluation
存在	presence	8	Awareness
喜ぶ	delight	8	Positive emotion
緊張	nervous	8	Negative emotion
動物	animal	8	Comparison/Metaphor
貰う	receive	7	Interaction
ストレス	stress	7	Negative emotion
遊び	play	7	Activity/Engagement
部屋	room	6	Environment
会話	conversation	6	Communication
増える	increase	6	Change
反応	reaction	6	Response
癒し	healing	6	Emotional support
笑い	laughter	6	Positive expression
ペット	pet	6	Comparison/Metaphor
触れる	touch	6	Physical interaction
気分	mood	5	Emotional state
元気	lively	5	Positive state
気持ち	feeling	5	Emotional state
接する	interact	5	Social interaction
最初	first	5	Temporal

Japanese	English Translation	Frequency	Semantic Category
友達	friend	5	Social relationship
多い	many	5	Quantity
上げる	raise	5	Action
きっかけ	catalyst	5	Facilitation
寝る	rest	5	Activity
早い	early	5	Temporal
名前	name	5	Identity
呼ぶ	call	5	Communication
大人	adult	5	Person
寂しい	lonely	5	Negative emotion
保護	protection	5	Care
優しい	gentle	5	Positive evaluation
撫でる	pet	5	Physical interaction
和らぐ	alleviate	4	Emotional improvement
自身	self	4	Identity
慣れる	get used to	4	Adaptation
世話	care	4	Activity
繋がる	connect	4	Social connection
助かる	supportive	4	Positive evaluation
ままごと	pretend play	4	Activity/Play
ありがたい	grateful	4	Positive emotion
相手	companion	4	Social relationship
短い	short	4	Temporal
リラックス	relax	4	Positive state
保育	childcare	3	Context
自ら	voluntarily	3	Agency
自宅	home	3	Environment
ごはん	meal	3	Daily activity
関わり	engagement	3	Engagement
軽減	relief	3	Improvement

Japanese	English Translation	Frequency	Semantic Category
長い	long	3	Temporal
愛着	bond	3	Emotional bond
沸く	emerge	3	Emergence
初め	initial	3	Temporal
恥ずかしい	shy	3	Emotion
離れる	leave	3	Separation
目的	purpose	3	Intention
おかげ	thanks to	3	Attribution
通る	pass through	3	Movement
安心	relief	3	Positive emotion
向かう	approach	3	Movement
見付ける	discover	3	Discovery
真っ先	immediately	3	Priority
本当	really	3	Emphasis
欲しい	want	3	Desire

Total: 91 translation pairs

Translation verification process:

1. Initial translations were generated based on context-appropriate meanings
2. All translations were reviewed by 2 bilingual researchers (AI: native Japanese speaker with English proficiency; HY: bilingual researcher)
3. Semantic equivalence was prioritized over literal translation
4. Terms with multiple possible meanings were translated according to their dominant usage in the corpus

Minimum threshold: All terms appeared in 3 or more documents with 3 or more total occurrences.
