# Supplementary material for: Impact of a Social Robot on Hospitalized Children, Caregivers, and Health Care Staff: Exploratory Observational Study
Source: JMIR Pediatr Parent. 2026 Jul 10;9:e93897. doi: 10.2196/93897 (PMC13352968; doi:10.2196/93897)
Supplement: Multimedia Appendix 4 [file pediatrics-v9-e93897-s004.pdf]

Multimedia Appendix 4. Statistical Results

Table S1. Detailed Statistical Results of Caregiver-Reported Outcomes (n=110)

One-sample tests comparing Likert scale ratings against baseline (3 = no change). All items used Wilcoxon signed-rank tests based on Shapiro-Wilk normality testing. P values were corrected using the Benjamini-Hochberg false discovery rate (FDR) procedure.

Item	Group	N	Mean (SD)	Median [IQR]	Mean Difference (95% CI)	Cohen d	P (raw)	P (FDR)
1. Smile frequency	Children	109	4.28 (0.67)	4.0 [1.0]	4.16-4.41	1.92	<.001	<.001
2. Play with peers	Children	107	3.28 (0.53)	3.0 [0.0]	3.18-3.38	0.53	<.001	<.001
3. Communication with staff	Children	108	3.56 (0.65)	3.0 [1.0]	3.43-3.68	0.86	<.001	<.001
4. Physical activity	Children	108	3.69 (0.77)	4.0 [1.0]	3.54-3.83	0.89	<.001	<.001
5. Stress/anxiety	Children	109	4.17 (0.76)	4.0 [1.0]	4.03-4.32	1.55	<.001	<.001
6. Enjoyment	Children	109	4.47 (0.62)	5.0 [1.0]	4.35-4.59	2.38	<.001	<.001
7. Adaptation to hospital life	Children	106	4.10 (0.70)	4.0 [1.0]	3.97-4.24	1.57	<.001	<.001
8. Communication with child	Caregivers	107	4.06 (0.71)	4.0 [1.0]	3.92-4.19	1.48	<.001	<.001
9. Communication with staff	Caregivers	107	3.64 (0.64)	4.0 [1.0]	3.51-3.76	1.00	<.001	<.001
10. Own stress/anxiety	Caregivers	107	4.02 (0.67)	4.0 [0.0]	3.89-4.15	1.51	<.001	<.001

Table S2. Results of Subgroup Analyses

Between-group comparisons of children's outcomes (caregiver-reported). Within-group comparisons tested ratings against baseline (3 = no change) using Wilcoxon signed-rank tests. Between-group comparisons used Mann-Whitney U tests with Benjamini-Hochberg FDR correction.

Age Group (≤5 years vs ≥6 years)

**Outcome**	**n (G1)**	**Mean (SD) (G1)**	**Cohen d (G1)**	**n (G2)**	**Mean (SD) (G2)**	**Cohen d (G2)**	**P (between)**
Adaptation to hospital life	62	4.05 (0.64)	1.64	44	4.18 (0.79)	1.50	.48
Child stress/anxiety	62	4.03 (0.72)	1.43	47	4.36 (0.76)	1.78	.04
Communication with staff	62	3.56 (0.67)	0.84	46	3.54 (0.62)	0.87	.96
Enjoyment of hospital life	62	4.31 (0.64)	2.03	47	4.68 (0.52)	3.26	.01
Physical activity	62	3.65 (0.73)	0.89	46	3.74 (0.83)	0.89	.48
Play with peers	61	3.25 (0.51)	0.49	46	3.33 (0.56)	0.58	.48
Smile frequency	62	4.15 (0.74)	1.54	47	4.47 (0.50)	2.91	.04

Hospital Stay Duration (≤1 week vs >1 week)

**Outcome**	**n (G1)**	**Mean (SD) (G1)**	**Cohen d (G1)**	**n (G2)**	**Mean (SD) (G2)**	**Cohen d (G2)**	**P (between)**
Adaptation to hospital life	70	4.14 (0.71)	1.61	32	3.97 (0.65)	1.50	.40
Child stress/anxiety	72	4.26 (0.71)	1.78	33	3.97 (0.81)	1.20	.37
Communication with staff	71	3.51 (0.63)	0.81	33	3.64 (0.70)	0.91	.54
Enjoyment of hospital life	72	4.53 (0.56)	2.75	33	4.30 (0.73)	1.79	.37
Physical activity	71	3.69 (0.77)	0.90	33	3.67 (0.78)	0.86	.81
Play with peers	70	3.27 (0.51)	0.53	33	3.33 (0.60)	0.56	.81
Smile frequency	72	4.33 (0.65)	2.05	33	4.12 (0.70)	1.61	.37

Child Sex (Female vs Male)

Outcome	n (G1)	Mean (SD) (G1)	Cohen d (G1)	n (G2)	Mean (SD) (G2)	Cohen d (G2)	P (between)
Adaptation to hospital life	54	4.19 (0.70)	1.69	52	4.02 (0.70)	1.46	.52
Child stress/anxiety	56	4.21 (0.85)	1.43	53	4.13 (0.65)	1.74	.57
Communication with staff	55	3.58 (0.69)	0.85	53	3.53 (0.61)	0.87	.86
Enjoyment of hospital life	56	4.50 (0.54)	2.78	53	4.43 (0.69)	2.07	.86
Physical activity	56	3.75 (0.79)	0.95	52	3.62 (0.75)	0.83	.71
Play with peers	55	3.22 (0.50)	0.44	52	3.35 (0.56)	0.62	.52
Smile frequency	56	4.39 (0.76)	1.84	53	4.17 (0.55)	2.14	.08

Playroom Use Frequency (High [at least once per day] vs Low-medium [less than once per day])

Outcome	n (G1)	Mean (SD) (G1)	Cohen d (G1)	n (G2)	Mean (SD) (G2)	Cohen d (G2)	P (between)
Adaptation to hospital life	64	4.16 (0.72)	1.61	37	4.03 (0.69)	1.50	.99
Child stress/anxiety	65	4.17 (0.74)	1.58	39	4.18 (0.82)	1.43	.99
Communication with staff	64	3.59 (0.68)	0.87	39	3.49 (0.56)	0.88	.99
Enjoyment of hospital life	65	4.49 (0.59)	2.53	39	4.44 (0.68)	2.11	.99
Physical activity	65	3.66 (0.82)	0.81	39	3.69 (0.73)	0.95	.99
Play with peers	64	3.27 (0.51)	0.52	38	3.26 (0.50)	0.52	.99
Smile frequency	65	4.29 (0.72)	1.79	39	4.26 (0.59)	2.11	.99

Table S3. Detailed Statistical Results of Staff-Reported Outcomes (n=32)

One-sample tests comparing Likert scale ratings against baseline (3 = no change). All items used Wilcoxon signed-rank tests. P values were corrected using the Benjamini-Hochberg FDR procedure.

Item No	Item	Group	N	Mean (SD)	Mean Difference (95% CI)	Cohen d	P (raw)	P (FDR)
1	Children's stress/anxiety	Children and Caregivers	32	4.19 (0.64)	3.96-4.42	1.84	<.001	<.001
2	Children's adaptation	Children and Caregivers	32	4.28 (0.63)	4.05-4.51	2.02	<.001	<.001
3	Overall impact on children	Children and Caregivers	32	4.62 (0.61)	4.41-4.84	2.67	<.001	<.001
4	Caregivers' stress/anxiety	Children and Caregivers	32	3.84 (0.63)	3.62-4.07	1.34	<.001	<.001
5	Overall impact on caregivers	Children and Caregivers	32	4.09 (0.89)	3.77-4.42	1.22	<.001	<.001
6	Communication with children	Children and Caregivers	32	3.94 (0.72)	3.68-4.20	1.31	<.001	<.001
7	Communication with caregivers	Children and Caregivers	32	3.72 (0.63)	3.49-3.95	1.13	<.001	<.001
8	Own stress/anxiety	Staff and Ward	32	3.38 (0.75)	3.10-3.65	0.50	.015	.016
9	Other staff's stress/anxiety	Staff and Ward	32	3.47 (0.67)	3.23-3.71	0.70	.001	.001
10	Communication with other staff	Staff and Ward	32	3.47 (0.57)	3.26-3.67	0.83	<.001	<.001
11	Ward atmosphere	Staff and Ward	32	4.50 (0.62)	4.28-4.72	2.41	<.001	<.001
12	Workload change	Staff and Ward	32	3.09 (0.53)	2.90-3.28	0.18	.37	.37

Notes:

- All within-group comparisons showed P<.001 (FDR-corrected) unless otherwise noted
- Effect sizes interpreted as small (0.2), medium (0.5), large (0.8), very large (1.2), and huge (≥2.0) [Sawilowsky, 2009]
- Between-group P values are FDR-corrected across all comparisons within each subgroup variable
- G1 = first group listed in each comparison; G2 = second group
