# Supplementary material for: Impact of a Social Robot on Hospitalized Children, Caregivers, and Health Care Staff: Exploratory Observational Study
Source: JMIR Pediatr Parent. 2026 Jul 10;9:e93897. doi: 10.2196/93897 (PMC13352968; doi:10.2196/93897)
Supplement: Checklist 1 [file pediatrics-v9-e93897-s005.pdf]

# Checklist 1. STROBE Statement—Checklist of Items for Observational Studies

**Study Title:** Impact of a Social Robot on Hospitalized Children, Caregivers, and Health Care Staff: Exploratory Observational Study

| Item No | Section/Topic | Checklist Item | Reported on Page/Section |
|---|---|---|---|
| **Title and Abstract** | | | |
| 1 | Title and abstract | (a) Indicate the study's design with a commonly used term in the title or the abstract | Title, Abstract |
| | | (b) Provide in the abstract an informative and balanced summary of what was done and what was found | Abstract |
| **Introduction** | | | |
| 2 | Background/rationale | Explain the scientific background and rationale for the investigation being reported | Introduction, paragraphs 1-4 |
| 3 | Objectives | State specific objectives, including any prespecified hypotheses | Introduction, paragraph 5 |
| **Methods** | | | |
| 4 | Study design | Present key elements of study design early in the paper | Methods: Study Design and Setting |
| 5 | Setting | Describe the setting, locations, and relevant dates, including periods of recruitment, exposure, follow-up, and data collection | Methods: Study Design and Setting |
| 6 | Participants | (a) Give the eligibility criteria, and the sources and methods of selection of participants | Methods: Participants |
| 7 | Variables | Clearly define all outcomes, exposures, predictors, potential confounders, and effect modifiers | Methods: Data Collection |

| Item No | Section/Topic | Checklist Item | Reported on Page/Section |
|---|---|---|---|
| 8 | Data sources/measurement | For each variable of interest, give sources of data and details of methods of assessment (measurement). Describe comparability of assessment methods if there is more than one group | Methods: Data Collection; Multimedia Appendix 2, Tables S1-S2 |
| 9 | Bias | Describe any efforts to address potential sources of bias | Discussion: Limitations |
| 10 | Study size | Explain how the study size was arrived at | Methods: Participants |
| 11 | Quantitative variables | Explain how quantitative variables were handled in the analyses. If applicable, describe which groupings were chosen and why | Methods: Statistical Analysis |
| 12 | Statistical methods | (a) Describe all statistical methods, including those used to control for confounding | Methods: Statistical Analysis; Multimedia Appendix 1 |
| | | (b) Describe any methods used to examine subgroups and interactions | Methods: Statistical Analysis |
| | | (c) Explain how missing data were addressed | Methods: Statistical Analysis |
| | | (d) If applicable, describe analytical methods taking account of sampling strategy | N/A (convenience sampling) |
| | | (e) Describe any sensitivity analyses | N/A |
| **Results** | | | |
| 13 | Participants | (a) Report numbers of individuals at each stage of study—eg numbers potentially eligible, examined for eligibility, confirmed eligible, included in the study, completing follow-up, and analysed | Results: Participant Characteristics; Figure 1B |
| | | (b) Give reasons for non-participation at each stage | Methods: Participants |

| Item No | Section/Topic | Checklist Item | Reported on Page/Section |
|---|---|---|---|
| | | (c) Consider use of a flow diagram | Figure 1B |
| 14 | Descriptive data | (a) Give characteristics of study participants (eg demographic, clinical, social) and information on exposures and potential confounders | Results: Participant Characteristics; Table 1 |
| | | (b) Indicate number of participants with missing data for each variable of interest | Table 1; Multimedia Appendix 4 |
| 15 | Outcome data | Report numbers of outcome events or summary measures | Results: Impact on Children and Caregivers; Health Care Staff Perspectives |
| 16 | Main results | (a) Give unadjusted estimates and, if applicable, confounder-adjusted estimates and their precision (eg, 95% confidence interval). Make clear which confounders were adjusted for and why they were included | Results: all subsections; Figures 2-4; Multimedia Appendix 4, Tables S1 and S3 |
| | | (b) Report category boundaries when continuous variables were categorized | Methods: Statistical Analysis (subgroup definitions) |
| | | (c) If relevant, consider translating estimates of relative risk into absolute risk for a meaningful time period | N/A |
| 17 | Other analyses | Report other analyses done—eg analyses of subgroups and interactions, and sensitivity analyses | Results: Consistency Across Patient Characteristics; Multimedia Appendix 4, Table S2 |
| **Discussion** | | | |
| 18 | Key results | Summarise key results with reference to study objectives | Discussion: Principal Results |

| Item No | Section/Topic | Checklist Item | Reported on Page/Section |
|---|---|---|---|
| 19 | Limitations | Discuss limitations of the study, taking into account sources of potential bias or imprecision. Discuss both direction and magnitude of any potential bias | Discussion: Limitations |
| 20 | Interpretation | Give a cautious overall interpretation of results considering objectives, limitations, multiplicity of analyses, results from similar studies, and other relevant evidence | Discussion: Comparison with Prior Work; Conclusions |
| 21 | Generalisability | Discuss the generalisability (external validity) of the study results | Discussion: Limitations |
| **Other Information** | | | |
| 22 | Funding | Give the source of funding and the role of the funders for the present study and, if applicable, for the original study on which the present article is based | Funding |

**Notes:**

- This checklist is based on the STROBE Statement (von Elm et al, 2007)
- N/A = Not applicable to this study design
- Page numbers should be updated after final formatting

**Reference:** von Elm E, Altman DG, Egger M, Pocock SJ, Gotzsche PC, Vandenbroucke JP; STROBE Initiative. The Strengthening the Reporting of Observational Studies in Epidemiology (STROBE) statement: guidelines for reporting observational studies. *Lancet*. 2007;370(9596):1453-1457.
